# Supplementary material for: Batch-to-Batch Variation and Patient Heterogeneity in Thymoglobulin Binding and Specificity: One Size Does Not Fit All
Source: J Clin Med. 2025 Jan 10;14(2):422. doi: 10.3390/jcm14020422 (PMC11765605; doi:10.3390/jcm14020422)
Supplement: Supplementary file 1 [file jcm-14-00422-s001.zip › jcm-3367187-supplementary.pdf]

Batch-to-batch variation and patient heterogeneity in Thymoglobulin binding and specificity: one size does not fit all

Nicoline H.M. den Hollander, Diahann T.S.L. Jansen Bart O. Roep

Supplementary data

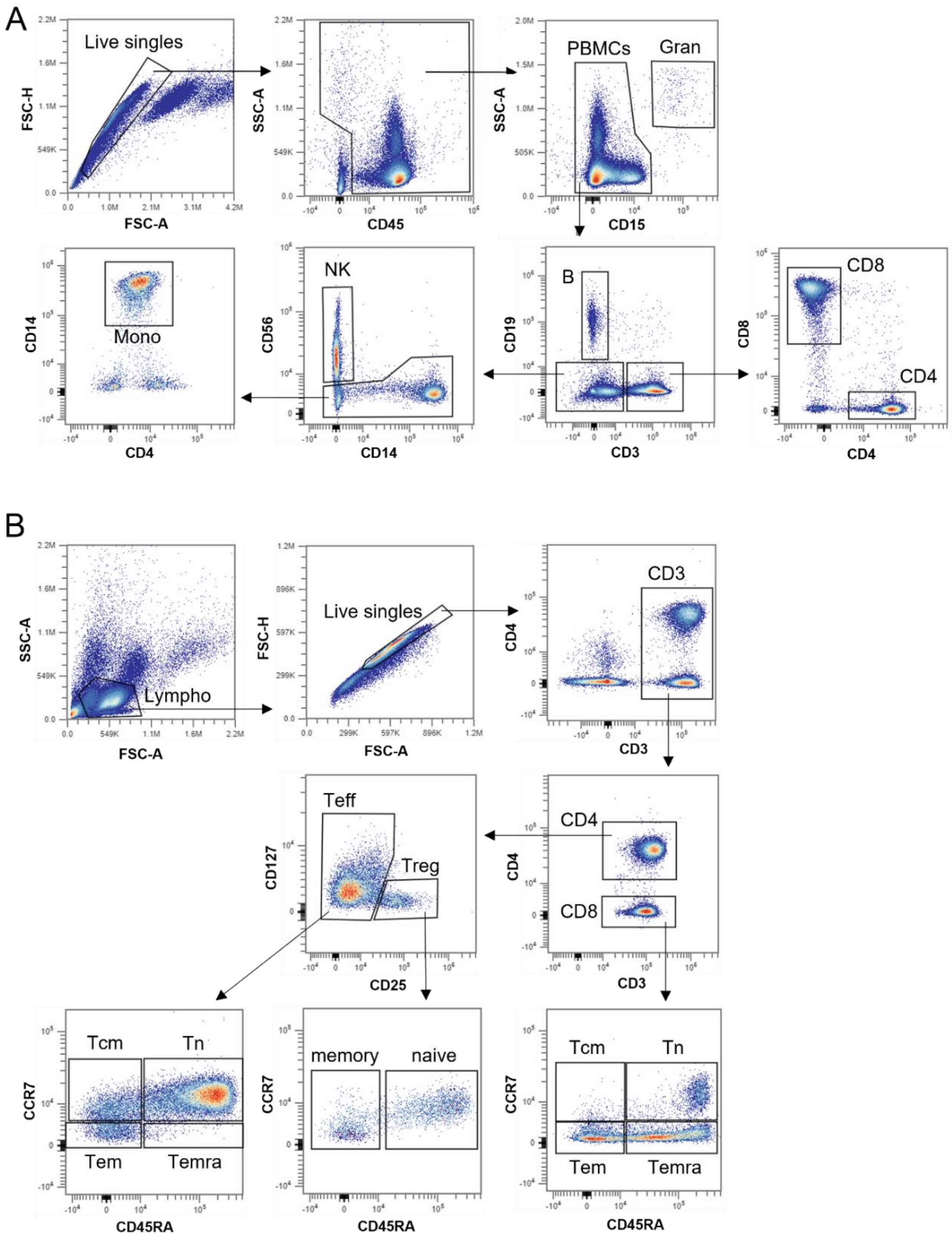

Figure S1. Gating strategy Schematic representation of flow cytometry gating strategy to identify PBMC subsets (**A**) and T cell subsets (**B**). *(Gran = granulocytes, NK = natural killer cells, Mono = monocyte, Lympho = lymphocyte, Teff = effector T cell, Treg = regulatory T cell, Tn = naive T cell, Tcm = central memory T cell, Tem = effector memory T cell, Temra = exhausted memory T cell).*

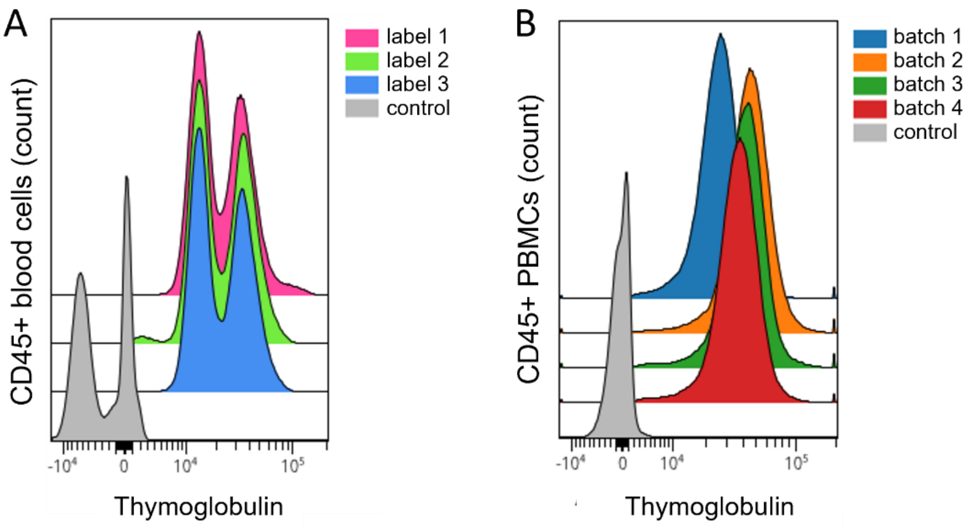

Figure S2. Conjugation efficiency and labeling of CD45⁺ cells by different Thymoglobulin batches
Thymoglobulin binding signal on CD45⁺ blood cells from three conjugation procedures following the same protocol and performed on the same batch. The samples are derived from one donor (**A**). Thymoglobulin binding signal on CD45⁺ PBMCs from four different batches of Thymoglobulin. Samples are stained with Thymoglobulin, followed by the PBMC panel antibodies. Control samples are only stained with panel antibodies (without Thymoglobulin). Each batch represents samples from the same five donors that are investigated in this study (**B**).

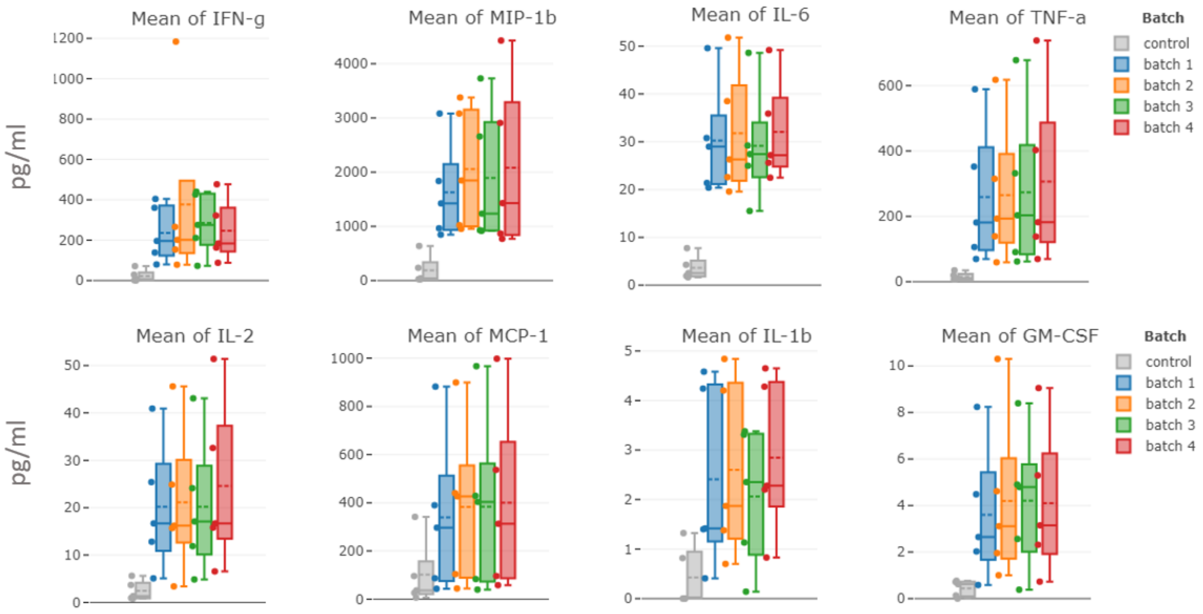

Figure S3. Cytokine serum levels after *in vitro* Thymoglobulin incubation of different batches
Absolute cytokine serum levels after Thymoglobulin incubation with blood from five donors are presented for each batch. Cytokine release syndrome (CRS)-related cytokines are presented in the upper four graphs.
